# The influence of preoperative determinants on quality of life, functioning and pain after total knee and hip replacement: a pooled analysis of Dutch cohorts

**DOI:** 10.1186/s12891-018-1991-0

**Published:** 2018-03-02

**Authors:** Stefanie N. Hofstede, Maaike G. J. Gademan, Theo Stijnen, Rob G. H. H. Nelissen, Perla J. Marang- van de Mheen, on behalf of the ARGON-OPTIMA study group, S. M. A. Bierma-Zeinstra, M. van Dijk, S. Kaarsemaker, P. M. van Kampen, P. A. Nolte, R. W. Poolman, Y. Pronk, M. Reijman, M. Stevens, B. J. W. Thomassen, S. H. M. Verdegaal, T. P. M. Vliet Vlieland

**Affiliations:** 1 Department of Orthopaedics, Leiden University Medical Center, Leiden, The Netherlands; 2 Department of Clinical Epidemiology, Leiden University Medical Center, Leiden, The Netherlands; 3 Department of Medical Statistics, Leiden University Medical Center, Leiden, The Netherlands; 4 Medical Decision Making, Department of Biomedical Data Sciences, Leiden University Medical Center, J10-S, PO Box 9600, 2300 RC Leiden, The Netherlands

**Keywords:** Total hip replacement, Total knee replacement, Preoperative, Postoperative, Quality of life, Functioning, Pain

## Abstract

**Background:**

Previous research has identified preoperative determinants that predict health related quality of life (HRQoL), functioning and pain after total knee or hip arthroplasty (TKA/THA), but these differed between studies and had opposite directions. This may be due to lack of power and not adjusting for confounders. The present study aims to identify the preoperative determinants that influence health related quality of life (HRQoL), functioning and pain after total knee or hip arthroplasty (TKA/THA).

**Methods:**

We pooled individual patient from 20 cohorts with OA patients data (*n* = 1783 TKA and *n* = 2400 THA) in the Netherlands. We examined the influence of age, gender, BMI and preoperative values of HRQoL, functioning and pain on postoperative status and total improvement. Linear mixed models were used to estimate the effect of each preoperative variable on a particular outcome for each cohort separately. These effects were pooled across cohorts using a random effects model.

**Results:**

For each increase in preoperative point in HRQoL, the postoperative HRQoL increased by 0.51 points in TKA and 0.37 points in THA (SF-36 scale). Similarly, each point increase in preoperative functioning, resulted in a higher postoperative functioning of 0.31 (TKA) and 0.21 (THA) points (KOOS/HOOS-ADL scale). For pain this was 0.18 (TKA) and 0.15 (THA) points higher (KOOS/HOOS-pain scale) (higher means less pain). Even though patients with better preoperative values achieved better postoperative outcomes, their improvement was smaller. Women and patients with a higher BMI had more pain after a TKA and THA. Higher age and higher BMI was associated with lower postoperative HRQoL and functioning and more pain after a THA.

**Conclusions:**

Patients with a better preoperative health status have better outcomes, but less improvement. Even though the independent effects may seem small, combined results of preoperative variables may result in larger effects on postoperative outcomes.

## Background

Total knee or hip arthroplasty (TKA/THA) is an effective treatment for most individuals who suffer from pain and loss of function due to end stage symptomatic osteoarthritis of the hip and knee (OA). In 2010, 109 and 153 patients per 100,000 persons received a TKA or THA respectively in Europe [1]. The development and progression of OA are strongly influenced by age and obesity and both occur more frequently in women [1]. Parallel to the rising prevalence of knee and hip OA, due to an ageing society and obesity, surgery rates are rising as well [2–4].

TKA and THA should not be performed too early since revision rates are higher in younger patients and the length of life of a prosthesis is limited [5]. On the other hand performing a surgery earlier gives more years of productive quality-adjusted life years (QALY’s). However, outcomes after revision surgery are generally worse compared to primary surgery [6]. Current practice shows that preoperative disease severity varies largely among centers and countries [7, 8], suggesting differences in timing. In addition, about 10–20% of the patients is not satisfied after primary TKA/THA [9–12], possibly caused by unmet expectations of patients or due to suboptimal timing of surgery.

Previous research has identified preoperative determinants that influence outcomes, but these differed between studies and had opposite directions [13]. This may be due to lack of power so that some studies did not find any effect, while other studies did not adjust for confounders. In addition, most registries collect a minimal data set [14] e.g. only the VAS scale for pain. Therefore, pooling the data from available cohort studies may provide more reliable evidence on which determinants influence the outcome after TKA/THA because of the larger sample size than separate studies and a more comprehensive set of questionnaires with the ability to measure each outcome more reliable compared to registry studies.

### Objective

The present study aims to examine the independent effect of several preoperative determinants for outcomes after TKA or THA by pooling individual patient data from available prospective cohorts in the Netherlands.

## Methods

### Study design and setting

The ARGON-OPTIMA (Outcome Predictors for TIMing of ArthropLasty) study is part of the ARGON program (Arthritis Research Group Orthopaedics in The Netherlands). Within this study, we pooled individual patient data from all available prospective TKA/THA cohorts in the Netherlands. All orthopaedic clinics in The Netherlands were invited to participate and submit data. We included prospective cohorts among patients with primary OA who underwent TKA or THA, with at least one preoperative and one postoperative measurement on functional or clinical outcomes and a follow-up of at least one year. Cohorts regarding metal-on-metal (MoM) prostheses were excluded, since these are not recommended in current guidelines in The Netherlands.

### Participants

Twenty hospitals submitted data and 20 cohorts from 11 hospitals were included. Nine hospitals were excluded because they did not meet the inclusion criteria. Of the included cohorts, 8 cohorts included 1783 knee OA patients undergoing primary TKA and 12 cohorts included 2400 hip OA patients undergoing primary THA. Table 1 shows the characteristics of patients per cohort.

**Table 1:** Description of included TKA and THA databases

Arthroplasty	Study	n	Females (%)	Age mean (SD)	BMI mean (SD)	Follow-up
TKA^a^	1	340	228 (67)	68.9 (9.3)	29.3 (7.6)	2 weeks, 3 months, 2–7 years
	2	382	271 (71)	67.0 (9.7)	29.5 (4.7)	1 year
	3	45	20 (44)	67.8 (6.5)	29.3 (5.1)	3, 6, 12 months
	4	101	66 (65)	68.9 (9.1)	30.9 (5.1)	6 weeks, 6, 12 months, 5 years
	5	496	274 (55)	65.9 (7.9)	27.6 (3.5)	6, 12, 24 months
	6	169	120 (71)	69.8 (9.9)	29.2 (4.7)	6 weeks, 3 months, 1 year
	7	41	22 (54)	62.2 (9.5)	32.0 (5.4)	3, 6 months, 4 years
	8	209	127 (61)	66.4 (10.2)	29.7 (6.4)	6 weeks, 3, 6, 12 months
THA^b^	1	498	319 (64)	65.7 (10.8)	26.9 (4.0)	2 weeks, 3 months, 2–7 years
	2	149	106 (71)	60.4 (6.9)	26.8 (4.2)	6 weeks, 3, 6, 12, 24 months
	3	398	247 (62)	66.6 (10.2)	27.2 (4.5)	1 year
	4	55	32 (58)	67.7 (9.7)	27.3 (3.6)	3, 6, 12 months
	5	73	46 (63)	65.2 (6.7)	28.0 (4.6)	6 weeks, 3, 6, 12, 24, 60 months
	6	26	18 (69)	62.9 (5.0)	24.5 (2.9)	6 weeks, 3, 6, 12 months
	7	354	228 (64)	65.9 (7.9)	26.4 (3.4)	3, 12 months
	8	100	58 (58)	68.7 (10.0)	28.2 (4.0)	6 weeks, 3, 12 months
	9	287	188 (66)	67.5 (10.6)	26.6 (4.1)	6 weeks, 3, 12 months
	10	73	46 (63)	66.7 (12.0)	26.5 (4.2)	3, 6, 12 months
	11	33	22 (67)	63.0 (11.9)	26.6 (4.3)	3, 6, 48 months
	12	354	257 (73)	69.0 (10.9)	28.2 (4.5)	6, 12, 24 months

^a^*TKA* Total Knee Artrhoplasty, ^b^*THA* Total Knee Arthroplasty

### Preoperative determinants

The assessed preoperative determinants were age, gender and BMIs. Furthermore, we examined the influence of preoperative health related quality of life (HRQoL), functioning and pain.

### Postoperative outcomes

We studied the effect on the absolute level of the postoperative outcome, but also on the extent of improvement to assess which patients would benefit most from change in health related quality of life (HRQoL), functioning and pain.

### Standardization

Since different cohorts used different questionnaires, these were standardized to compare the same domains across different questionnaires. Furthermore, multiple questionnaires were sometimes used to measure the same domain within a cohort. As each patient should be included only once for each domain, we ordered questionnaires in their ability to measure each outcome reliably. This was done during an ARGON consortium meeting. A group of experts within the ARGON consortium discussed about the ordering of questionnaires until consensus was reach. The following main points were taken into concern: is it a general or disease specific questionnaire, how many items are used to calculate the composite score, and is it a common used questionnaire in the Netherlands.

Only the highest rated questionnaire in each dataset was included. The following ordering was used:

- Health related quality of life:
  1. Physical component summary scale of the 36-item short form health survey (SF-36/RAND-36) (36 items)
  2. Physical component summary scale of the 12-item short form health survey (SF-12) (12 items)
  3. EuroQoL 5 Dimensions (EQ-5D) (5 items)
- Functioning:
  1. Hip/knee disability and Osteoarthritis Outcome Score (HOOS/ KOOS) subscale Activities of Daily Living (ADL) (17 items)
  2. Western Ontario & McMaster Universities Osteoarthritis Index (WOMAC) subscale Physical Function (PF) (17 items)
  3. HOOS-Short form (PS)/KOOS-Short form (PS) (5 items)
  4. Oxford Hip Score (OHS) subscale function (6 items)/ Oxford Knee Score (OKS) subscale function (5 items) according to Harris et al. [15, 16]
- Pain:
  1. HOOS/ KOOS subscale Pain (10 items)
  2. WOMAC subscale Pain (5 items)
  3. OHS subscale Pain (6 items)/ OKS subscale Pain (7 items) according to Harris et al. [15, 16]
  4. Visual Analogue Scale (VAS) pain scale

For each patient we calculated the standardized score at each time point using the following formula (functioning as example):

$$ \mathrm{Standardized}\ \mathrm{Functioning}\ \mathrm{score}\ \mathrm{for}\ \mathrm{patient}\ \mathrm{X}\ \left(\mathrm{at}\ \mathrm{t}\mathrm{ime}\ \mathrm{point}\ \mathrm{t}\right)=\frac{\left(\mathrm{functioning}\ \mathrm{score}\ \mathrm{for}\ \mathrm{patient}\ \mathrm{X}\ \mathrm{in}\ \mathrm{cohort}\ \mathrm{Y}\ \left(\mathrm{at}\ \mathrm{t}\mathrm{ime}\ \mathrm{point}\ \mathrm{t}\right)\hbox{--} \mathrm{preoperative}\ \mathrm{mean}\ \mathrm{of}\ \mathrm{functioning}\ \mathrm{among}\ \mathrm{patient}\mathrm{s}\ \mathrm{in}\ \mathrm{cohort}\ \mathrm{Y}\right)\ }{\mathrm{preoperative}\ \mathrm{SD}\ \mathrm{of}\ \mathrm{functioning}} $$

Some questionnaires differed in the direction of the scale e.g. on the VAS pain scale, lower scores mean less pain whereas lower scores mean more pain on the HOOS/KOOS subscale pain. The direction of all scales were recoded so that higher scores referred to better values).

### Statistical analysis

Data of TKA and THA were analyzed separately. As a first step, linear mixed models (LMM) were used to estimate the influence of each preoperative variable on each major outcome for each cohort separately, adjusted for the other variables. As determinants were included in the fixed part of the LMM: the standardized preoperative score (HRQoL, functioning and pain), age, sex, BMI and follow-up time. Interaction terms were fitted between the determinants and follow-up time. In the LMM the patients were specified as the subjects, with an unstructured covariance matrix. This was done for each standardized postoperative outcome. In the second step, the regression coefficients from all cohorts were pooled using a random effects model to obtain one pooled estimate for each preoperative variable and outcome. Given the pooled estimates of the impact of preoperative status on postoperative status, we can also determine the total improvement (postoperative minus the preoperative status). If patients would have the same amount of improvement, 1 point higher in preoperative status would result in a postoperative status of 1 point higher. So if the increase in postoperative status is < 1 (e.g. 0.4), this means that the improvement is 0.6 points smaller for every point increase in preoperative status.

Given that preoperative scores were standardized, the pooled regression coefficient should be interpreted as the number of standard deviations that an outcome will change, per point increase in the preoperative variable. For example looking at the effect of age on postoperative functioning with a standardized regression coefficient of 0.2 and the preoperative SD of functioning is 7, this means that one year increase in age is estimated to increase the postoperative functioning by: 0.2*7. To facilitate interpretation of the pooled standardized regression coefficients of age, BMI and gender, we transformed standardized regression coefficients back to a 0–100 scale (e.g. HOOS, SF-36), using the preoperative standard deviation (SD) of the study with the highest weight in the random effects model. In addition, we will illustrate the potential size of the effects by describing scenarios.

SPSS 20 was used to perform the LLM and Stata 11.1 for the random effects model. A *p*-value of 0.05 was considered significant in all analyses.

### Assessment of heterogeneity

The I^2^ statistic was used to quantify heterogeneity between cohorts. This can be interpreted as the percentage of total variability in a set of effect sizes due to between-studies variability. We considered results as heterogeneous when I^2^ was 50% or greater [17].

### Ethics approval and consent to participate

The Medical Ethical Committee of the Leiden University Medical Center (CME P15.043/SH/sh) confirmed on February 13 2015 that ethical approval for this type of study is not required under the Dutch Medical Research (Human Subjects) Act. The hospitals that supplied anonymous data obtained written informed consent from the study participants.

## Results

### Age, gender and BMI

Table 2 shows the pooled estimates of the effect of age, gender and BMI on outcomes as well as the transformed values. Most effects were small and homogeneous. For TKA, only gender and BMI were significantly associated with pain. Women had more pain postoperatively than men (4 points lower on a 0–100 scale, where 100 is no pain). An increase in BMI with one point, resulted in more postoperative pain (0.5 points on a 0–100 scale). For THA, age and BMI were significantly associated with HRQoL, functioning and pain. One year increase in age decreased postoperative functioning by 0.3 point on a 0–100 scale. Furthermore, women perceived more pain after a THA (2 points on a 0–100 scale).

**Table 2:** The influence of patients characteristics on postoperative outcomes after TKA and THA

Arthroplasty	Patients characteristic	Outcome	Cohorts (n)	Patients (n)	Standardized regression coefficients (95% CI)	Transformed regression coefficient (0–100 scale)	I^2^ (%)
TKA^a^	Age	HRQoL^d^	4	774	0.00 (−0.00, 0.01)	0.00	0.0
		Functioning	6	1021	− 0.01 (− 0.01, 0.00)	− 0.18	0.0
		Pain	6	1102	0.01 (− 0.00, 0.02)	0.16	47.0
	Gender (women)	HRQoL	4	774	−0.05 (− 0.23, 0.13)	− 0.38	0.0
		Functioning	6	1021	−0.24 (− 0.50, 0.01)	−4.12	53.6
		Pain	6	1102	−0.25 (− 0.50, − 0.01)	−3.92	50.5
	BMI^c^	HRQoL	4	774	−0.02 (− 0.06, 0.02)	−0.23	76.1
		Functioning	6	1021	−0.01 (− 0.05, 0.02)	−0.18	62.5
		Pain	6	1102	−0.03 (− 0.05, − 0.01)	−0.47	13.1
THA^b^	Age	HRQoL	8	1436	−0.01 (− 0.02, − 0.01)	−0.08	0.0
		Functioning	10	1271	−0.02 (− 0.02, − 0.01)	−0.33	0.0
		Pain	10	1492	−0.01 (− 0.01, − 0.00)	−0.18	0.0
	Gender (women)	HRQoL	8	1436	−0.10 (− 0.22, 0.01)	−0.78	0.0
		Functioning	10	1271	−0.11 (− 0.22, 0.01)	−1.95	10.9
		Pain	10	1492	−0.11 (− 0.21, − 0.00)	−2.00	0.0
	BMI	HRQoL	8	1436	−0.03 (− 0.04, − 0.01)	−0.23	0.0
		Functioning	10	1271	−0.02 (− 0.04, − 0.01)	−0.35	0.0
		Pain	10	1492	−0.02 (− 0.03, − 0.00)	−0.36	0.0

^a^*TKA* Total Knee Arthroplasty, ^b^*THA* Total Hip Artrhoplasty, ^c^*BMI* Body Mass Index, ^d^*HRQoL* Health Related Quality of Life

### Health related quality of life

**Fig. 1:**
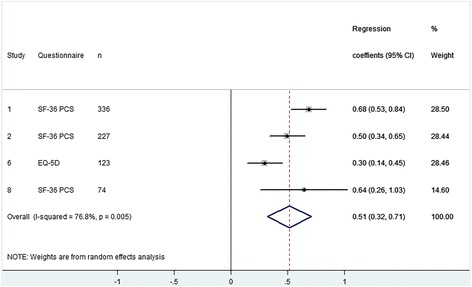
Forest plot - The influence of preoperative HRQoL on postoperative HRQoL after TKA

**Fig. 2:**
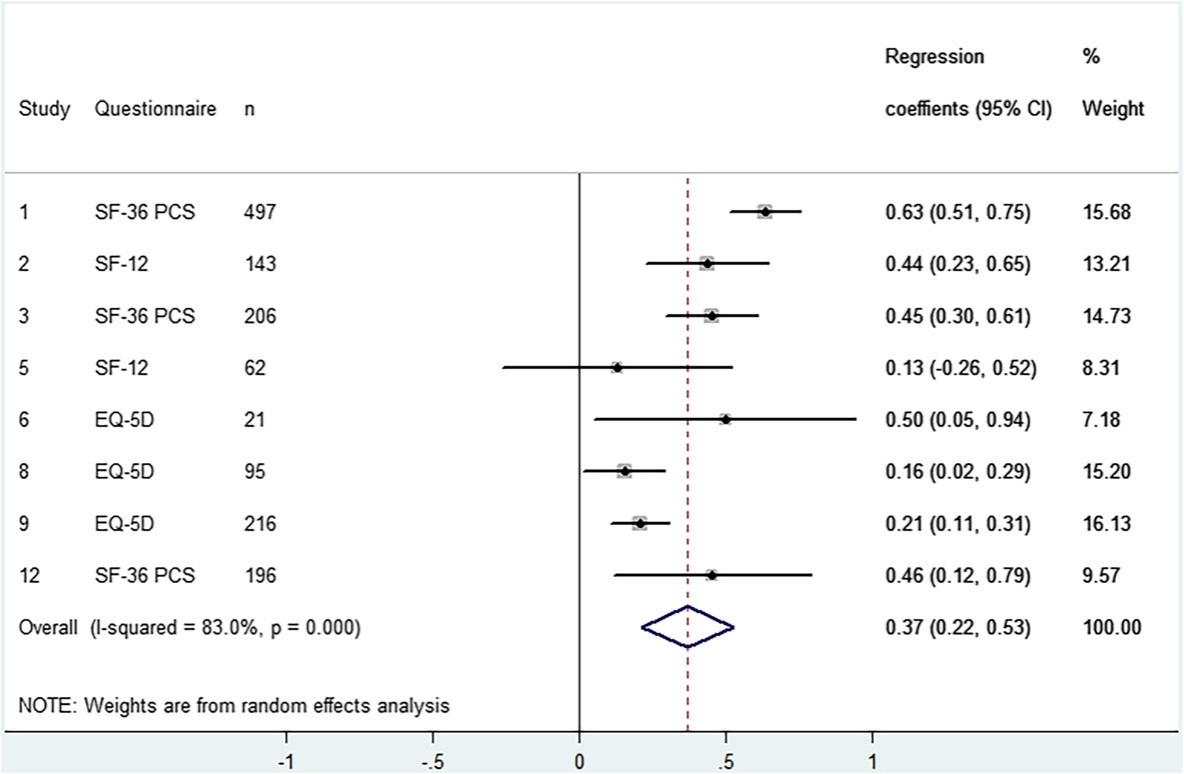
Forest plot - The influence of preoperative HRQoL on postoperative HRQoL after THA

Four cohorts examined the effect of preoperative HRQoL on postoperative HRQoL in 760 patients after TKA (Fig. 1). Eight cohorts examined this effect in 1436 patients with a THA (Fig. 2). A significant positive effect of preoperative HRQoL was found of 0.51 (95% CI 0.32 to 0.71) for patients after TKA and 0.37 (95% CI 0.21 to 0.53) after THA. This means that a patient with 1 point higher preoperative HRQoL on average achieves a 0.51 point (TKA) and 0.37 point (THA) higher postoperative HRQoL on the SF-36 scale. At the same time, if patients with a 1 point higher preoperative HRQoL reach a 0.51 point higher postoperative HRQoL after TKA, this also means that their improvement is 0.49 (0.51–1) points less. For THA this implies 0.63 (0.37–1) points less improvement postoperative. The results were heterogeneous, meaning that included cohorts differed with respect to the estimated effect for either TKA or THA.

### Functioning

**Fig. 3:**
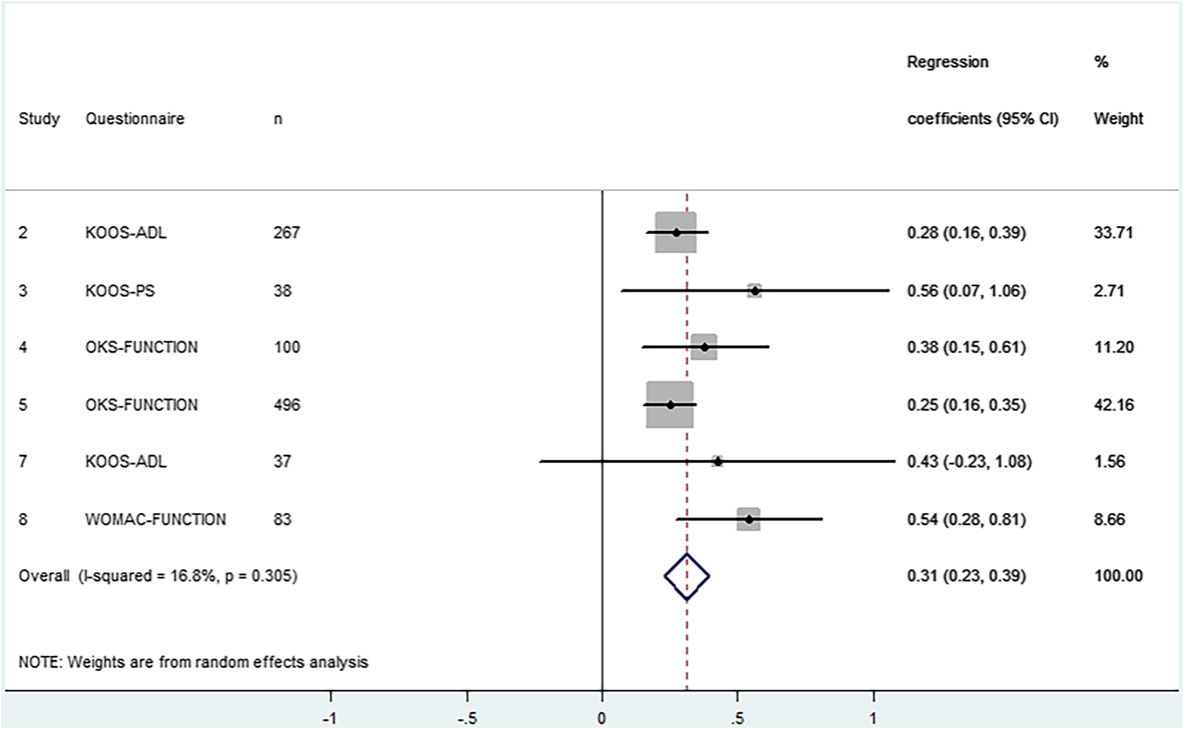
Forest plot - The influence of preoperative functioning on postoperative functioning after TKA

**Fig. 4:**
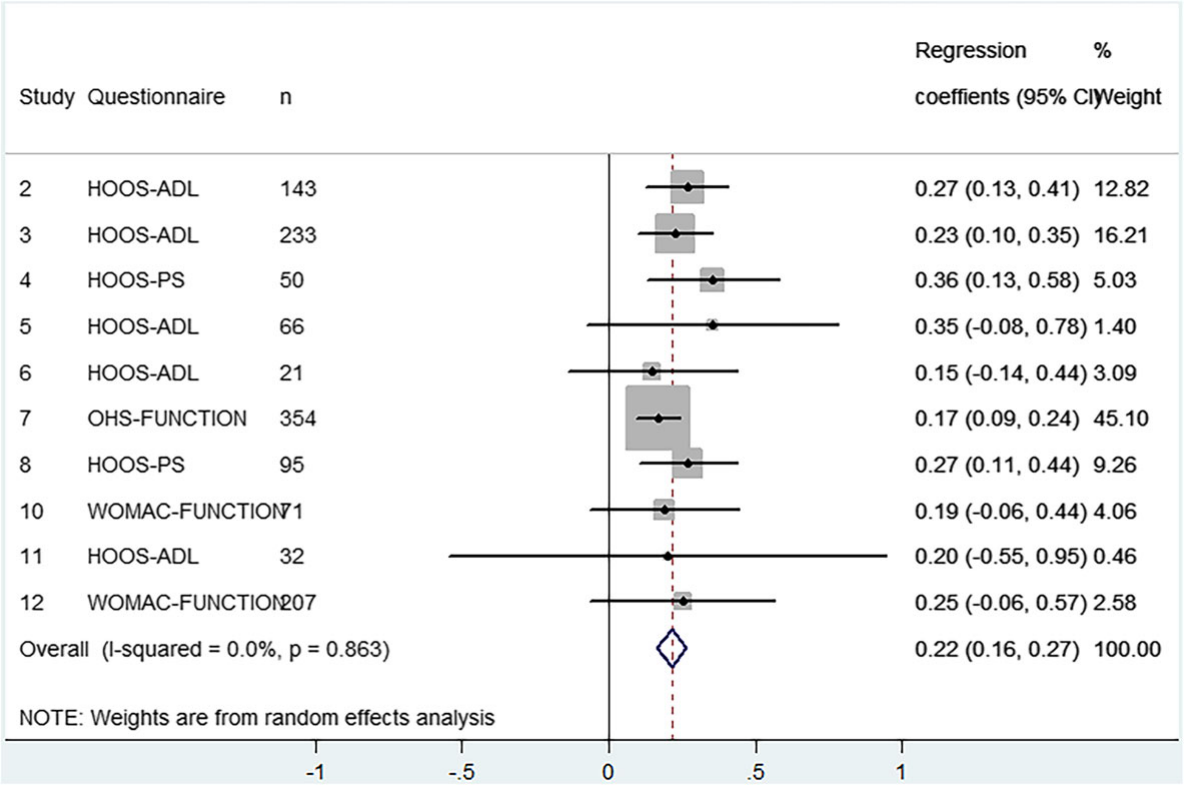
Forest plot - The influence of preoperative functioning on postoperative functioning after THA

Six cohorts examined the effect of preoperative functioning on postoperative functioning in 1021 patients with a TKA (Fig. 3) and 10 cohorts examined this effect in 1271 patients with a THA (Fig. 4). We found a significant positive effect of 0.31 (95% CI 0.23 to 0.39) for TKA and 0.21 (95% CI 0.16 to 0.26) for THA. This means that a patient with a 1 point higher preoperative functioning on average achieves a 0.31 points higher postoperative functioning on the KOOS scale (TKA) and 0.21 points of the HOOS scale (THA). At the same time this means that these patients have a 0.69 and 0.79 point less improvement for TKA and THA respectively for every 1 point higher on preoperative functioning. The results were homogeneous meaning that the estimated effects did not differ between cohorts.

### Pain

**Fig. 5:**
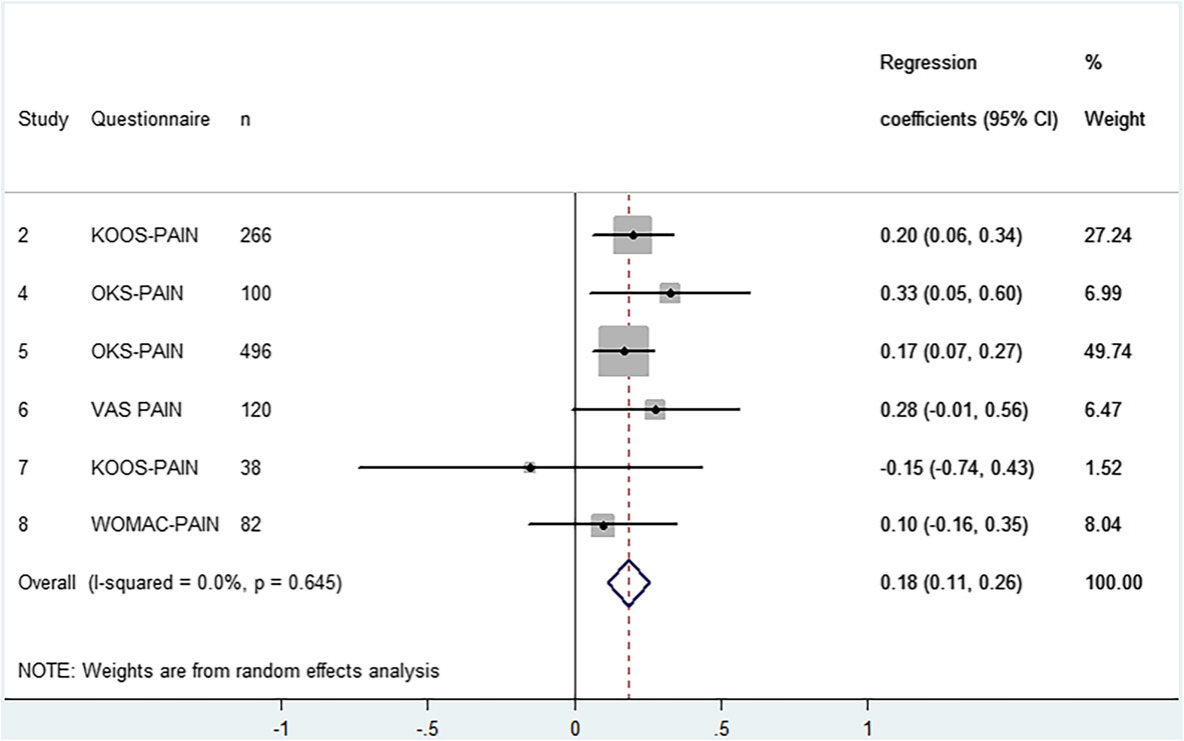
Forest plot - The influence of preoperative pain on postoperative pain after TKA

**Fig. 6:**
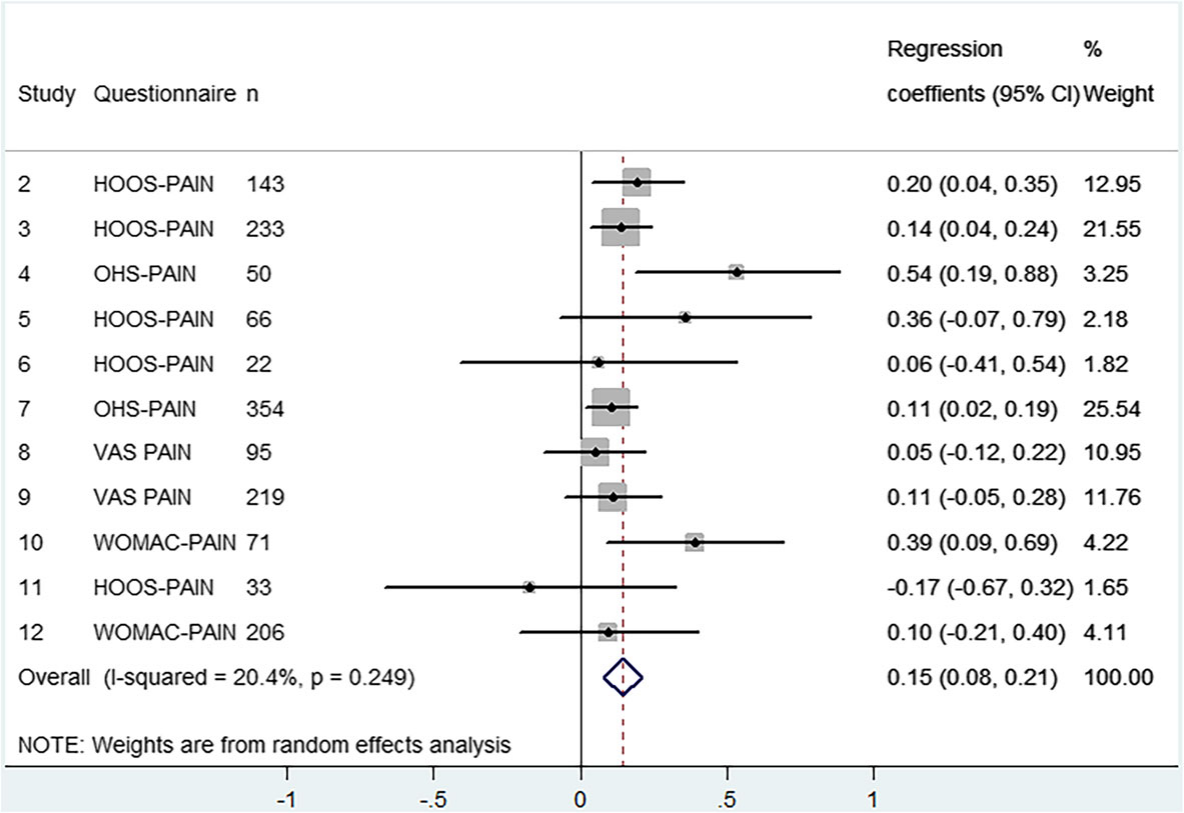
Forest plot - The influence of preoperative pain on postoperative pain after THA

Six cohorts examined the effect of preoperative pain on postoperative pain in 1102 TKA patients (Fig. 5) and 12 cohorts examined this effect in 1492 THA patients (Fig. 6). We found that every point increase in preoperative pain (i.e. less pain) was associated with 0.18 (95% CI 0.11 to 0.26) point increase in postoperative pain after a TKA and 0.15 (95% CI 0.08 to 0.21) after a THA. This also means that patients with less preoperative pain improve 0.82 points less after TKA and 0.85 points less after THA. The results were homogeneous meaning that the estimated effects did not differ between cohorts.

### Combined results

Even though the independent effect of one variable may be small, the combined effect of different determinants may result in clinically relevant differences. Table 3 shows some hypothetical scenarios in which several determinants are combined. The first scenario is that a patient first loses some weight and reduces the BMI with 5 points to improve the postoperative functioning after THA. This takes some time (e.g. 5 years) and a higher age decreases the postoperative functioning. Suppose that due to the weight loss the preoperative functioning increases with 5 points (on a 0–100 scale). Taken together, this results in a 1.2 points higher postoperative outcome. The second scenario is that a surgeon thinks a patient is too young to perform a THA. If a patient receives this THA 10 years later, and during this 10 years the patient also gains weight due to an inactive lifestyle (e.g. 10 points of BMI) and the functioning also reduces with 10 points (on a 0–100 scale), his/her postoperative functioning will be 9 points lower compared to the situation if she/he had received THA surgery 10 years earlier. The effect of these scenarios on HRQoL and pain are also shown in Table 3. Overall effects vary between 1.2 and 6.5 points better postoperative outcomes for scenario 1 and between 1.6 and 9 points worse postoperative outcomes for scenario 2.

**Table 3:** Hypothetical examples of combined data within scenarios

Arthroplasty	Assessed outcome	Effect of age	Effect of BMI	Effect of preoperative status	Total effect on postoperative outcome (points)^a^
Scenario 1: A patient loses weight (X points) and increases preoperative status by Y points, this takes Z years					
*X, Y, Z = 5 (*e.g. *in 5 years BMI decreases from 30 to 25, KOOS HRQoL/ functioning/ pain increases from 35 to 40)*					
TKA	HRQoL	0	0	5*0.51	2.6
	Functioning	0	0	5*0.31	1.6
	Pain	0	5*0.47	5*0.18	3.3
THA	HRQoL	5*−0.08	5*0.23	5*0.37	2.6
	Functioning	5*−0.33	5*0.35	5*0.22	1.2
	Pain	5*−0.18	5*0.36	5*0.15	1.7
*X, Y, Z = 10 (*e.g. *in 10 years BMI decreases from 35 to 25, KOOS HRQoL/ functioning/ pain increases from 35 to 45)*					
TKA	HRQoL	0	0	10*0.51	5.1
	Functioning	0	0	10*0.31	3.1
	Pain	0	10*0.47	10*0.18	6.5
THA	HRQoL	10*−0.08	10*0.23	10*0.37	5.2
	Functioning	10*−0.33	10*0.35	10*0.22	2.4
	Pain	10*−0.18	10*0.36	10*0.15	3.3
Scenario 2: A patient gains weight (X points) and decreases preoperative status by Y points, this takes Z years					
*X, Y, Z = 5 (*e.g. *in 5 years BMI increases from 25 to 30, HOOS HRQoL/ functioning/ pain decreases from 40 to 35)*					
TKA	HRQoL	0	0	5*−0.51	−2.6
	Functioning	0	0	5*−0.31	−1.6
	Pain	0	5*−0.47	5*−0.18	−3.3
THA	HRQoL	5*−0.08	5*−0.23	5*−0.37	−3.4
	Functioning	5*−0.33	5*−0.35	5*−0.22	−4.5
	Pain	5*−0.18	5*−0.36	5*−0.15	−3.5
*X, Y, Z = 10 (*e.g. *in 10 years BMI increases from 25 to 35, HOOS HRQoL/ functioning/ pain decreases from 45 to 35)*					
TKA	HRQoL	0	0	10*−0.51	−5.1
	Functioning	0	0	10*−0.31	−3.1
	Pain	0	10*−0.47	10*−0.18	−6.5
THA	HRQoL	10*−0.08	10*−0.23	10*−0.37	−6.8
	Functioning	10*−0.33	10*−0.35	10*−0.22	−9.0
	Pain	10*−0.18	10*−0.36	10*−0.15	−6.9

^a^On a 0–100 scale

## Discussion

The present pooled analysis of 1783 knee and 2400 hip OA patients shows that patients with a higher preoperative quality of life or functioning and less pain also have better postoperative outcomes but that they improve less. Furthermore, women and patients with a higher BMI had more postoperative pain and less improvement after both TKA and THA. Higher age and higher BMI was associated with lower postoperative HRQoL and functioning and more pain after a THA. However, preoperative quality of life, functioning and pain seem to be most consistently associated with outcomes after both TKA and THA.

Our results regarding the effect of preoperative status on outcomes are consistent with other studies that also found that patients with worse preoperative functioning had greater improvements [18–21], but did not achieve the postoperative level of those with higher preoperative functioning [22–28]. Contrary, other studies showed opposite results regarding the direction and size of the effect of age, gender and BMI. Santaguida et al. [29] performed a systematic review about patient characteristics affecting the prognosis after TKA/THA and concluded that an older age is related to worse functioning, but that age and sex do not influence postoperative pain level. We found that women had more pain after a TKA (4 points on a 100 point scale) and THA (2 points on a 100 point scale), even though this may not be a clinically relevant difference [30]. For TKA no association with age or gender and functioning was found. In addition, a previous review about prognostic determinants in THA reported that preoperative functioning was most consistently associated with better outcomes [13]. In addition, another systematic review on preoperative predictors on outcomes in THA [31] concluded that only patients’ poor preoperative functioning affects the outcome after THA. This was also found for patients with a TKA [32, 33]. Consistent with our finding, Lingard et al. [33] found that patients with severe pain had worse outcomes after a TKA. Other studies also identified other determinants, such as radiological scores, severity of inflammations or comorbidities. A disadvantage of using multiple cohorts with different protocols for data acquisition was that we could not include these determinants. The linear mixed model had to be the equal for each cohort, so that regression coefficients in each cohort have the same meaning. Thus the prognostic determinants found in this present study are not exhaustive; there may be other determinants that have an additional effect on the outcome.

The effect of different preoperative determinants on the postoperative outcomes after TKA and THA may seem to be small on itself, but if taken together they may add up to a clinically relevant effect. However, the scenarios should be interpreted with care, because these are hypothetical examples based on observational data and cannot be interpreted causally. The overall effects of the virtual scenarios which were calculated as examples vary between 1.2 and 6.5 points better postoperative outcomes and between 1.6 and 9 points worse postoperative outcomes. These scenarios provide more insights how small differences may add up or cancel each other out. This probably explains why most effects do not reach a clinically significant difference. Usually a 10% difference (i.e. 10 points on a 0–100 scale [30]) is considered as clinically relevant, but is a 10% difference the right criterion? Postoperative TKA/THA scores increases on average by 20–40 points on a 0–100 scale (results not shown) compared to preoperative scores regardless of the preoperative status. Thus is it realistic to use a difference of 10 points to define whether it is clinically relevant to operate now or wait, based on differences in preoperative determinants?

It is important to realize that the effects found in our study are not only the effect of the surgery, but also the effect of regression to the mean (RTM). RTM occurs because values are observed with random error, such as random fluctuations in a subject [34]. This means that patients with low preoperative scores are more likely to have higher scores during the next measurement and that patients with high preoperative scores are more likely to have lower scores during the next measurement, even without surgery. This results on average in a larger “improvement” for patients with lower preoperative scores compared to patients with higher baseline scores. Although different methods have been proposed to estimate the size of the RTM effect, but no solution is available to distinguish the real change due to surgery from the change due to RTM. Furthermore, we had to standardize different questionnaires measuring the same domain. Ideally, a minimal dataset should be composed, so that is more easily comparable without the need of standardization since standardized regression coefficients are more difficult to interpret [35]. A strength of our study is that we pooled existing cohort studies. Most of these studies collected a comprehensive set of questionnaires. Although national arthroplasty registries are established, these registries differ from clinical studies. Most registries focus on long-term data collection and therefore focus on minimal data sets and collect patient and operative information, but not all registries collect patient-reported outcomes [36]. If registries collect patient-reported outcomes such as HRQoL, function or pain most often short questionnaires are used, with only 1 or 2 questions covering the domain e.g. VAS-scale for pain or the EQ-5D to measure HRQoL. Most of the in our study included cohort studies used more comprehensive questionnaires with the ability to measure each outcome more reliable. On the other hand using questionnaires with composite scores has some weaknesses. Different patients may have very different domain scores but these may still result in the same composite score. In our study we therefore used domain scores of different questionnaires (functioning and pain) besides the overall HRQoL composite score, which may reduce this problem. Another potential problem is that there may be cultural differences between countries in how questionnaires are answered, but this would only influence our results if these cultural differences would affect e.g. elderly patients differently than younger patients thereby resulting in a different relationship of age with the outcomes.

## Conclusion

The information regarding the combined effects of preoperative determinants on postoperative outcomes will support orthopaedic surgeons to estimate differences in outcome after a joint replacement for specific patient groups, i.e. poorer outcomes for patients with a worse preoperative status, but with greater postoperative improvement compared to patients with higher preoperative scores. In addition, preoperative status may decline during a long surgical delay period and thereby lead to worse postoperative outcomes if no other non-surgical treatments are started. On the other hand, it may sometimes be better to first optimize the patient’s preoperative condition or to reduce for example their BMI. The present study may support orthopaedic surgeons in their decision making by giving an estimate of the magnitude of the effect for different scenarios. Future studies should combine the results of our study with observational cohort studies among OA patients who did not have surgery yet, specific survival data from medical literature and the effects on survival of the artificial joint to assess optimal timing of surgery. This is needed to assess the long-term impact for the patient of the decision to perform surgery at a certain preoperative state of specific patient groups.

## Abbreviations

ADL: Activities of Daily Living
BMI: Body Mass Index
CI: Confidence interval
EQ-5D: EuroQoL 5 Dimensions
HOOS: Hip disability and Osteoarthritis Outcome Score
HRQoL: Health Related Quality of Life
KOOS: Knee disability and Osteoarthritis Outcome Score
LMM: Linear Mixed Models
MoM: Metal-on-metal
OA: Osteoarthritis
OHS: Oxford Hip Score
OKS: Oxford Knee Score
PF: Physical Function
SF-12: 12-item short form health survey
SF-36: 36-item short form health survey
THA: Total Hip Artrhoplasty
TKA: Total Knee Arthroplasty
VAS: Visual Analogue Scale
WOMAC: Western Ontario & McMaster Universities Osteoarthritis Index
